# Cultural diffusion dynamics depend on behavioural production rules

**DOI:** 10.1098/rspb.2022.1001

**Published:** 2022-08-10

**Authors:** Michael Chimento, Brendan J. Barrett, Anne Kandler, Lucy M. Aplin

**Affiliations:** ^1^ Cognitive and Cultural Ecology Research Group, Max Planck Institute of Animal Behavior, Am Obstberg 1, Radolfzell 78315, Germany; ^2^ Department for the Ecology of Animal Societies, Max Planck Institute of Animal Behavior, Am Obstberg 1, Radolfzell 78315, Germany; ^3^ Centre for the Advanced Study of Collective Behaviour, University of Konstanz, Universitätsstraße 10, Konstanz 78464, Germany; ^4^ Department of Biology, University of Konstanz, Universitätsstraße 10, Konstanz 78464, Germany; ^5^ Department of Human Behavior, Ecology and Culture, Max Planck Institute for Evolutionary Anthropology, Deutscher Platz 6, Leipzig 04103, Germany; ^6^ Division of Ecology and Evolution, Research School of Biology, The Australian National University, 46 Sullivan Creek Road, Canberra, Australian Capital Territory 2600, Australia

**Keywords:** cultural evolution, social learning, agent-based model, network-based diffusion analysis, experience weighted attraction models, reinforcement learning‌

## Abstract

Culture is an outcome of both the acquisition of knowledge about behaviour through social transmission, and its subsequent production by individuals. Acquisition and production are often discussed or modelled interchangeably, yet to date no study has explored the consequences of their interaction for cultural diffusions. We present a generative model that integrates the two, and ask how variation in production rules might influence diffusion dynamics. Agents make behavioural choices that change as they learn from their productions. Their repertoires may also change, and the acquisition of behaviour is conditioned on its frequency. We analyse the diffusion of a novel behaviour through social networks, yielding generalizable predictions of how individual-level behavioural production rules influence population-level diffusion dynamics. We then investigate how linking acquisition and production might affect the performance of two commonly used inferential models for social learning; network-based diffusion analysis, and experience-weighted attraction models. We find that the influence that production rules have on diffusion dynamics has consequences for how inferential methods are applied to empirical data. Our model illuminates the differences between social learning and social influence, demonstrates the overlooked role of reinforcement learning in cultural diffusions, and allows for clearer discussions about social learning strategies.

## 1. Introduction

Cultural evolution, or the changes over time in distributions of the types, forms or functions of socially learned traits, provides a secondary inheritance system that potentially enables rapid adaptive plasticity [1–4]. Social learning, the process that underlies cultural evolution, has a well-accepted definition of learning that occurs through observations of the behaviour, or interactions with the products of behaviour, of others [5]. It is perhaps well accepted owing to its ambiguity, as it encompasses many different phenomena. In particular, social learning may refer to (1) the learning of a novel skill from conspecifics, such as a cockatoo learning to open bins from associates [6], or (2) the influence that social information exerts upon behavioural choice, such as when a stickleback fish chooses feeders surrounded by more conspecifics [7]. In the first case, learning describes an event of acquisition of knowledge about a new behaviour as a result of social transmission [8]. In the second case, learning describes the changes in weights given to known behaviours when producing a behaviour, owing to social influence (i.e. response facilitation or contagion [9,10]). This ambiguity is further complicated by the circular relationship between these two phenomena. Production of a behaviour requires acquisition of knowledge of that behaviour, and acquisition requires observable productions. This relationship is captured in the definition of social transmission: when a behaviour is produced by an individual, it exerts a positive causal influence on the rate at which that individual’s associates acquire the same behaviour [8]. The conceptual distinction of origin (acquisition) and maintenance (production) of behaviour was previously highlighted by Galef [11] in the debate over why social learning is adaptive. Galef identified one critical cognitive factor that influences production directly: reinforcement learning, which he hypothesized influenced the maintenance or abandonment of cultural traits [11]. However, little is understood about how cultural dynamics are influenced by variation in such learning rules.

The breadth of the term ‘social learning’, and the circular relationship between acquisition and production, can also lead to ambiguity over the precise target of causal factors that influence cultural evolution. Generally, acquisition depends on cognitive [12–14], social [8,15,16] and environmental factors [17,18]. These factors include how individuals bias attention during learning [14], association patterns and network structure [8,19], and resource availability or habitat fragmentation [20]. These factors can also influence realized production behaviour [18,21], *but not necessarily in the same way as acquisition*. For example, there may be imprecision over the target of social learning biases or strategies—do they act upon the process of acquisition or production, or both? This has led to potential for miscommunication, for example in the long-standing debates over definitions of conformity [22]. Here, we aim to clarify the relationship between acquisition and production. We develop a simulation framework that treats these concepts separately, but permits their interaction via a process of social transmission [8]. Our mathematical model builds upon Galef’s verbal model, provides new predictions for how changes to learning rules of production influence diffusion dynamics, and offers clarity in discussions of the targets of social learning biases, strategies or constraints.

Acquisition and production have been modelled separately as approaches to two central goals of empirical studies of animal culture. One goal has been to identify when social transmission is responsible for the diffusion, or spread, of a behaviour through a population, as opposed to asocial innovation. A primary determinant of social transmission is the opportunity for naive individuals to learn from others, represented by their social association. This correlation between association and social transmission is at the core of network-based diffusion analysis (NBDA) [8,16,23], which has been used to identify social learning (*sensu* social transmission) in birds [6,24–26], fish [27,28], cetaceans [29–31] and primates [32–35]. Another goal is the identification of social influence, or how individuals integrate social information during decision-making between behaviours [12,36]. This has been studied using experience-weighted attraction (EWA) models—an extended reinforcement learning model [37] originally developed to understand how behavioural choices change over time in economic games [12,38,39]. Owing to their flexibility, EWA models are a popular method to identify social learning (*sensu* social influence on production). EWA has been used to study social learning strategies, theoretically [40,41] and in humans [12,42–44] and non-human animals [4,45–47].

As with any model-based inferential framework, both NBDA and EWA have shortcomings. NBDA assumes that the order of acquisition is correlated to network structure in cases of social transmission. However, acquisition of knowledge is not directly observable without behavioural output. Thus, NBDA assumes that the order of first observed production of a behaviour *is equivalent* to the order of acquisition. Divergences between acquisition of knowledge and first-productions might spell trouble for this assumption. Secondly, NBDA analyses do not usually account for behavioural frequency information (although recommended [16,48], see [24,49,50]). Regarding EWA, its formalization requires the definition of a set repertoire of behaviours that an individual may produce. To date, implementations have not accounted for differences in repertoire size over time or between individuals, and thus are restricted to situations where there are no innovations or diffusion, as individuals must have homogeneous repertoires. In summary, the dynamics of cultural evolution are not fully captured by a model of acquisition that does not account for production, nor by a model of production with no change in repertoire. We must simultaneously account for potential acquisition of novel behaviour, and the complex decision-making that contributes to the maintenance of behaviour in order to describe dynamics of cultural change with some generality. Yet, beyond specialized models of language evolution [51–53], no general, species-agnostic model of cultural evolution exists in which both acquisition and production are connected.

Here, we develop a simulation model that unites both processes by adapting the dynamics of NBDA and EWA frameworks. First, we explore how changes to production rules might affect the diffusion of a novel behaviour through networks of agents. We uncover new relationships between production and diffusion dynamics, and find that certain production rules result in more or less divergent orders between acquisition and first-production events. However, inferential NBDA models are blind to such divergences, and EWA models are blind to any heterogeneity in repertoire. To understand the consequences of this for the practical application of inferential methods, we then apply inferential models of NBDA and EWA to data generated by our model. We generate agents’ acquisition and production times, and behavioural output under known underlying mechanisms, e.g. social transmission or asocial innovation, and feed these data into inferential NBDA and EWA models. We find that linking production and acquisition limits the inferential power of these two popular methods. Finally, we discuss how our framework helps clarify broader discussions of social learning biases and strategies.

## 2. Methods

Our model simulated both the diffusion of a novel behaviour through a population, and the agents’ decisions of which behaviours to produce throughout the diffusion. Throughout the paper, we use ‘diffusion’ to refer to the spread of knowledge of the novel behaviour through a population. ‘Acquisition’ is when an agent learned that it may produce the novel behaviour. ‘First-production’ is when an agent first performed it. Acquisition and production were implemented as two sub-models that are mathematically consistent with prior applications of inferential NBDA [16] and EWA [12] frameworks, respectively. Some conventional notation has been altered be more accessible. Inferential NBDA statistically tests for acquisition of behaviour via social transmission against a null hypothesis of asocial innovation. It estimates the plausibility of social transmission by accounting for association with knowledgeable individuals, and orders or times of acquisition by naive individuals. Inferential EWA is a dynamic learning model that uses time-series behavioural data to estimate how individuals balance social and personal information, as well as recent and past information, in decision-making. While there are many ways to formulate either model of acquisition or production, we have chosen to follow these specific frameworks to robustly evaluate how linking acquisition and production might affect the inferential value of either model.

Importantly, we transformed these inferential models into a generative model. We used their assumed dynamics to produce probabilities of acquisition or production of a novel behaviour—generating data using known parameters, rather than inferring parameters from observed data. We connected the two sub-models, allowing them to influence one another. The sub-model of acquisition determined agents’ repertoires, i.e. behaviours able to be produced, while the sub-model of production determined which behaviour was chosen for production from their repertoires in each timestep, providing behavioural frequency information. Those frequencies then influenced the acquisition probability of a novel behaviour by naive agents in the following timestep. Thus, our model’s output was the cultural dynamic generated by linked processes of acquisition and production, conditioned on specific parameter values of either sub-model. We also explored the dynamics of the sub-models of acquisition and production in isolation (detailed in electronic supplementary material, S1, figure S1).

We assumed a population of constant size *N* = 24. Agents were situated within a social association network that constrained observation and acquisition opportunities to connected nodes. We used a random regular network architecture, with fixed degree (*k* = 6) associates for each agent (electronic supplementary material, figure S2). Random regular architecture was chosen because degree was important to standardize between agents—it directly affected the dynamics of acquisition (detailed in equation (2.2)). Edge weights were set to 1 to eliminate stochastic variation arising from differences in association. We explored other network architectures (electronic supplementary material, text S2), but the primary focus of this paper was to understand how learning rules governing behavioural production, rather than social structure, influenced diffusion dynamics. Population size was not varied, but we expect that larger populations would lead to longer diffusions. However, it would not impact the results as only degree influences either sub-model.

At the beginning of a simulation, all agents initially had a repertoire of one behaviour, 'a', interpreted as an established tradition that had already diffused through the population. To remove stochasticity from differences in innovation timing, one randomly selected ‘seed’ agent also had knowledge of a novel behaviour 'b'. Both behaviours obtained an equal reward (*r*_*a*_ = *r*_b_ = 1) when performed. Each timestep, agents had the opportunity to expand their repertoire by acquiring behaviour b, if it had been produced by at least one of their associates (see §2a). Additionally, agents chose one behaviour from their repertoire to produce. Their choice was influenced by past personal experiences of rewards, as well as social information from their neighbours (see §2b). Here reinforcement learning played an important role: when an agent acquired knowledge of behaviour b, its expected value was initially 0, as agents had never personally experienced reward from the behaviour, and we assumed that agents did not attend to socially observed rewards. The value of the behaviour was learned only through individual experience of producing the behaviour (see equation (2.4)). This cycle of potential acquisition and production repeated until the novel behaviour b had been acquired and produced at least once by each agent.

### (a) Sub-model of behavioural acquisition

Each timestep, agents had the opportunity to acquire knowledge of novel behaviour b through social transmission. The probability of acquisition depended on an agent’s associates and their behavioural productions, a rate of social transmission, and a rate of asocial innovation. We estimated the probability of acquisition of behaviour b for each naive individual *i*, i.e. individuals that only possessed behaviour a, at each timestep *t* by calculating

2.1 $$P(\text{individual}\;i\;\text{acquires}\;b|t)=1-\exp(-\lambda_{b}(T_{i}(t)+A)).$$

The baseline learning rate (*λ*_b_) represented how easily the novel behaviour was learned, either socially or asocially. *A* represented the presence of asocial innovation, and could take a value of 0 or 1. The transmission function *T*_*i*_(*t*) recorded the relative usage of behaviour b of all knowledgeable associates of agent *i* over a memory window of *m* timesteps. The transmission function *T*_*i*_(*t*) was defined by

2.2 $$T_{i}(t)=T(a_{i},w_{b}(t))=s\sum_{\;j=1}^{N}a_{i,j}w_{b,j}(t),$$

whereby the sum was taken over agent *i*’s associates as *a*_*i*,*j*_ = 1 if *i* and *j* were connected in the association network and *a*_*i*,*j*_ = 0 otherwise. *w*_b,*j*_(*t*) defined the transmission weight of agent *j* representing the proportion of time this agent produced behaviour b within the last *m* timesteps

2.3 $$w_{b,j}(t)=\frac{\sum_{r=t-m}^{t}n_{b,j}(r)}{m}.$$

We set *n*_b,*j*_(*r*) = 1 if agent *j* produced b at timestep *r*; otherwise *n*_b,*j*_(*r*) = 0. Lastly, the sum was multiplied by *s*, the rate of social transmission. Higher values of *s* resulted in increased probability of transmission per knowledgeable associate, relative to the asocial innovation.

**Figure 1.**
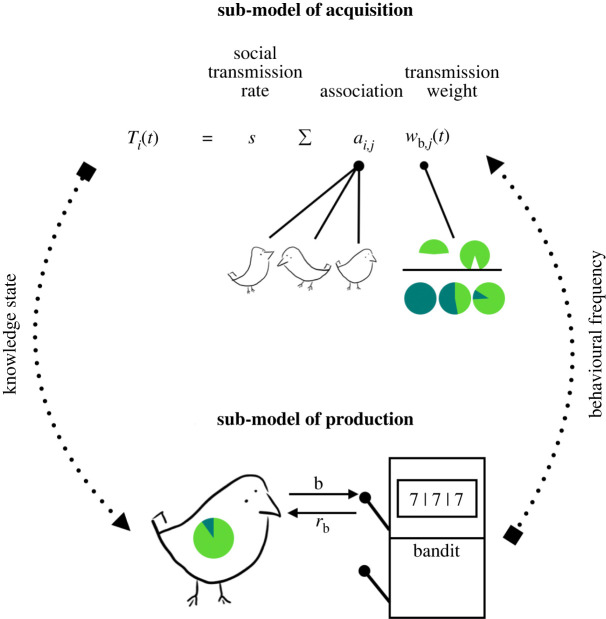
Model schematic. Each timestep, naive agents had the opportunity to acquire knowledge about novel behaviour b from their associates. Acquisition was conditioned on association and the frequencies that associates produced b, as well as a rate of social transmission, as defined by the transmission function *T*_*i*_. If an agent acquired b, it could then produce b using the sub-model of production. Production probabilities changed over time, depending on personal experience and social information. Behavioural frequency information from the production sub-model then informed acquisition probabilities in the following timestep. (Online version in colour.)

In summary, the transmission weight function was the nexus of our sub-models of acquisition and production, as illustrated by figure 1. It allowed behavioural frequencies, derived from the production sub-model detailed in the following section, to influence transmission probabilities calculated from the acquisition sub-model.

### (b) Sub-model of behavioural production

Each timestep, each agent *i* produced a behaviour k from its repertoire *Z*_*i*_ (consisting of either {*a*} only or {*a*, *b*}) with probability *P*_*i*,k_(*t*) as follows. Agents updated their expected values given their personal experience of producing a behaviour at *t* − 1 (equation (2.4)), which influenced the probability of producing either behaviour given personal information only (equation (2.5)). However, agents also considered social influence in their choice using a record of the observed productions of their associates (equation (2.6)). Changes in personal and social information potentially led to a changed production probability at time *t* (equation (2.7)), in a dynamic consistent with decision-biasing reinforcement learning [36]. Four parameters governed how these values changed with experience: recent experience bias (*ρ*), a risk-appetite bias (*α*), a social information bias (*σ*) and frequency-dependent production bias (*f*).

First, agent *i* calculated the expected value (*E*_*i*,k_(*t*)) for any behaviour *k* in its repertoire using the value function [37]

2.4 $$E_{i,k}(t)=\rho r_{k}(t-1)+(1-\rho )E_{i,k}(t-1).$$

The term *r*_k_(*t* − 1) described the reward obtained from behaviour *k* in the last timestep: if behaviour *k* has been produced *r*_k_(*t* − 1) = 1, otherwise *r*_k_(*t* − 1) = 0. This assumption allowed for information loss over time. In the first timestep, agents were initialized with *E*_*i*,a_(1) = 1. Once an agent acquired the novel behaviour, *E*_*i*,b_(*t*) = 0. For behaviour k, $\max(E_{i,k})=r_{k},\;\forall i$, meaning expected values could never exceed the true reward. The parameter *ρ* determined the importance of the most recently received reward *vis-à-vis* previously experienced rewards.

In the second step, agent *i* converted its expected value from the behaviours in its repertoire *Z*_*i*_ into a probability using a softmax rule

2.5 $$I_{i,k}(t)=\frac{\exp(\alpha^{-1}E_{i,k}(t))}{\sum_{k\in Z_{i}}\exp(\alpha^{-1}E_{i,k}(t))}.$$

The parameter *α* controlled the sensitivity towards differences between expected values: low values resulted in an almost deterministic choice of the highest expected value behaviour (risk-aversion), whereas high values led to choices almost independent from expected values. Equation (2.5) assigned probabilities to behaviours even when their expected value was 0, providing the possibility of production without prior experience.

Next, agent *i* evaluated its social information by counting how many times its associates had produced any of the behaviours in its repertoire within the memory window composed of the last *m* timesteps. This sliding window *m* allowed agents to forget social information over time, similar in function to how agents were allowed to forget personal information when non-chosen behaviours were updated with *r*_k_ = 0 in equation (2.4) above. The number of observations was then modulated by the frequency-dependent production bias parameter *f*, which determined the strength of frequency-dependent influence on the agent

2.6 $$S_{i,k}(t)=\frac{{\left(\sum_{\;j=1}^{N}a_{i,j}\sum_{r=t-m}^{t}n_{k,j}(r)\right)}^{\;f}}{\sum_{k\in Z_{i}}{\left(\sum_{\;j=1}^{N}a_{i,j}\sum_{r=t-m}^{t}n_{k,j}(r)\right)}^{\;f}}.$$

Agents only observed behaviours that were in their own repertoire. If an agent did not know how to produce a behaviour, its observation could not influence that agent’s choice in that timestep. However, this information did influence the potential acquisition of this behaviour as described in §2a.

Lastly, agent *i* combined its personally experienced rewards and socially observed behaviours to generate probabilities of producing the behaviours in its repertoire

2.7 $$P_{i,k}(t)=(1-\sigma )I_{i,k}(t)+\sigma S_{i,k}(t),$$

where *σ* described the preference for individual and social information. Values of *σ* close to 0 would almost neglect social information while values close to 1 would almost neglect individual information.

### (c) Conditions

In order to understand how production rules influenced diffusion dynamics, we explored diffusions across populations under a variety of parameter settings, summarized in table 1. Each combination of these parameters formed one constellation. For each constellation, we ran 100 simulations.

**Table 1.** Summary of tested parameters for the production sub-model. Mathematical symbol, name, values tested and short description of these values.

name	symbol	values	description of effect
social information bias	*σ*	$\in \left\{\begin{array}{c} 0.25\\ 0.05\\ 0.75 \end{array}\right\}$	mostly personal information influences agents
			personal and social information equally influence agents
			mostly social information influences agents
freq. dependent production bias	*f*	$\in \left\{\begin{array}{c} 0.33\\ 1\\ 3 \end{array}\right\}$	agents disproportionately weigh the minority behaviour
			agents linearly weigh observed behaviours
			agents disproportionately weigh the majority behaviour
memory window	*m*	$\in \left\{\begin{array}{c} 10\\ 20\\ 30 \end{array}\right\}$	agents remember *m* timesteps of observed behaviours
recent experience bias	*ρ*	$\in \left\{\begin{array}{c} 0.01\\ 0.10\\ 0.99 \end{array}\right\}$	agents learn slowly, preferring prior experience

			agents learn quickly, preferring recent experience
risk-appetite	*α*	$\in \left\{\begin{array}{c} 0.5\\ 1\\ 2 \end{array}\right\}$	agents are risk-adverse, seeking higher expected values
			agents are risk-neutral, weighing expected values proportionally
			agents are risk-tolerant, indifferent to expected values

In §3a, we used the following parameter constellation as reference:

2.8 σ=0.5,f=1,m=10,ρ=0.1andα=1.

We focused on results from the combinations of constellations described by table 1, using a sensitivity analysis to explore the effect of each parameter while holding all others at reference. Intermediary and extended values of these parameters were also evaluated (e.g. *σ* ∈ {0.125, 0.375, 0.625, 0.875}), although they showed no unexpected patterns and were thus excluded from the paper for concision. The effect of *ρ* on the evolution of expected values in equation (2.4) is nonlinear (i.e. the difference in effect between *ρ* = 0.013 and *ρ* = 0.014 is magnified compared with the difference in effect between *ρ* = 0.79 and *ρ* = 0.80); thus we tested three different orders of magnitude, rather than differences in linear magnitude. We also varied parameters related to the acquisition sub-model (see electronic supplementary material, text S2 for network structure, *λ*_b_, *s* and *A* values), but have excluded these from the main text, as the effect of network structure and social transmission on diffusion is a well-explored subject [19,54,55]. The baseline learning rate was set to *λ*_b_ = 0.05, the social transmission rate was set to *s* = 5. In order to isolate the effect of variation in production rules on diffusion via social transmission, and eliminate any variation due to random innovation events, the asocial innovation parameter was set to *A* = 0 for §3a. However, it is unrealistic to expect that no independent innovation occurs in natural diffusions. Thus, *A* = 1 in simulations used to test the performance of inferential models (see §2e).

### (d) Measurements

To describe the dynamics of the diffusion of variant *b* through the population, we calculated three quantities: tempo, divergence and delay. To quantify diffusion tempo, we recorded the timesteps at which each agent acquired the novel behaviour and first produced the novel behaviour. We also recorded behavioural frequencies every timestep. From these data, we derived time-to-diffusion (TTD), i.e. the time until the whole population had acquired behaviour b, intervals between each acquisition event, time-to-first-production (TTFP), i.e. the time until the whole population had produced behaviour b at least once, vectors of order of acquisition (***o***_a_), order of first-production (***o***_p_), time of acquisition (***t***_a_) and time of first-production (***t***_p_). Vectors ***o***_a_ and ***o***_p_ contained the position of each agent in the acquisition sequence and first-production sequence, respectively. Vectors

***t***_a_ and ***t***_p_ contained the time of acquisition and time of first-production by each agent, respectively.

To quantify divergence in the orders of acquisition and first-production, we calculated the mean Manhattan distance

2.9 $$d_{\mathrm{order}}=\frac{1}{N-1}\sum_{i=1}^{N}|o_{a}(i)-o_{p}(i)|$$

representing the mean difference between acquisition and first-production position in the population (excluding the first-production of the seed agent, i.e. the agent at position 1; thus *N* − 1, as ***o***_a_(1) − ***o***_p_(1) = 0 per definition). Additionally, we calculated the proportion of the population that obtained production positions that differed from their acquisition positions.

To quantify the delay between the acquisition and first-production timing, we used vectors ***t***_a_ and ***t***_p_ and calculated the mean difference between the times of acquisition and first-production

2.10 $$d_{\mathrm{time}}=\frac{1}{N}\sum_{i=1}^{N}|t_{a}(i)-t_{p}(i)|.$$

For each measurement, we report means and 92% percentile intervals in brackets.

### (e) Testing performance of NBDA and EWA on generated data

Lastly, we explored how NBDA and EWA might perform on data generated by our model. NBDA assumes that the order of first-production is equivalent to that of acquisition. Yet, when acquisition was conditioned on production by other individuals (as in our model) these orders could diverge. To test how this might affect the inference of the underlying generative process (i.e. social transmission or asocial innovation), we first created ‘ideal’ data generated using the pure NBDA dynamic with which we expected NBDA to provide strong support for the correct generative process. We then created two datasets of more realistic data that violated NBDA’s assumption that orders of acquisition and first-production were identical. To create ideal data, we recorded time-of-acquisition data, generated by either asocial innovation only (*A* = 1, *s* = 0), or social transmission (*A* = 1, *s* = 5) where the transmission weight was *w*_*j*,b_(*t*) = 1 if associate *j* was knowledgeable, otherwise 0, removing the influence of production on transmission. To create realistic data, we recorded time-of-first-production data, generated by either social transmission using transmission weights as we have defined them (equation (2.3)), or asocial innovation only. For each of these four scenarios, we simulated 10 diffusions at each parameter constellation (table 1).

We then ran inferential TADAc NBDA models [16] on each simulation’s data. For simulations with social transmission, the seed agent was included as a demonstrator. For realistic data, we included a transmission weight in the inferential models, defined as a production rate of the novel behaviour for each agent (total productions of *b* divided by timesteps knowledgeable). Using recommended inferential steps, we ran both a social and asocial TADAc models to determine support for social transmission [8]. We compared corrected Akaike information criterion (AICc) scores between the two models to determine relative support for social or asocial innovation [8]. Support for social transmission was defined as ΔAICc > 0, where ΔAICc = AICc_asocial_ − AICc_social_. We reported the median of all ΔAICcs per condition to get an idea of what the average support for the generative process might be.

We used a similar strategy to evaluate the performance of EWA analyses. We estimated only recent experience bias (*ρ*) and social information bias (*σ*) under three scenarios: (1) homogeneous repertoires and no diffusion, (2) heterogeneous repertoires with only social transmission (*A* = 0, *s* = 5) and (3) heterogeneous repertoires with only asocial innovation (*A* = 1, *s* = 0). Scenario 1 was a proof of concept with ideal data generated by using the pure EWA dynamic, and the EWA model was expected to recover accurate estimates. Scenarios 2 and 3 explored how EWA performed on data collected from populations with heterogeneous repertoires, when behaviours were actively diffusing or being innovated. For each scenario, we performed 10 simulations for each combination of *ρ* and *σ* values ({0.25, 0.5, 0.75} for both). All other parameters were set to reference.

For scenario 1, we recorded behavioural productions for 300 timesteps to provide sufficient power. In scenarios 2 and 3, each simulation ran for twice as long as its TTD (social transmission: range 65−199; asocial innovation: range 89−235 timesteps), thus recording equal numbers of observations during diffusion, and after all agents acquired knowledge of the novel behaviour. We then fitted inferential models to the production data from each simulation from these three scenarios using Hamiltonian Markov chain Monte Carlo (MCMC). Models were run using five chains, 4000 iterations, with 1000 warm-up iterations, with all estimates based on over 4000 effective samples from the posterior (range: 4952–10916). All models were fitted using R v. 4.0.2 [56] with Stan v. 2.27 [57] via Rstan v. 2.21.2 [58]. Good model convergence was confirmed from evaluating rank histograms and ensuring parameters had Gelman–Rubin’s statistic $\hat{R}\leq 1.01$ (range: 0.999–1.002) [59,60]. Additionally, we plotted priors against the posteriors to assess how well the models identified parameters. For each parameter, we report mean and 92% highest posterior density interval (HPDI), or the narrowest interval containing 92% of posterior samples.

## 3. Results

**Figure 2.**
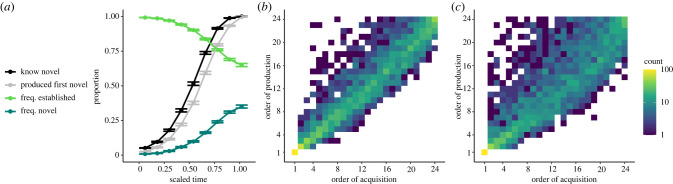
Acquisition and production dynamics throughout diffusion. Data presented from 100 simulations. (*a*) Average knowledge acquisition curve of novel behaviour (black), first-production curve of the novel behaviour (grey), and behavioural frequency data of the established behaviour (light green) and the novel behaviour (dark green) at reference setting. (*b*) Density plot comparing the order in which the novel behaviour entered an agent’s repertoire (*x*-axis) and the order in which that agent first produced the novel behaviour (*y*-axis) at reference setting. Each count is one agent from one simulation. Measuring the order of first-production usually did not reflect the order of acquisition, and only about 25% of all agents produced the novel behaviour in the same order that they acquired it. (*c*) Density plot for the parameter setting that obtained the most divergence (83.2% divergent agents; *σ* = 0.25, *f* = 3, *ρ* = 0.99, *a* = 0.5, *m* = 10).

Simulations with linked acquisition and production sub-models generated the dynamics depicted in figure 2*a*. At the reference parameter constellation (equation (2.8), henceforth reference), knowledge of the novel behaviour diffused to all individuals in 54.22 [37.96, 76.28] timesteps (mean TTD [92% PI]). The first-production curve lagged behind the knowledge acquisition curve, with all agents producing the novel behaviour at least once in 61.11 [44.96, 85.04] timesteps (mean TTFP). Agents retained a higher expected value for the established behaviour and still preferred its usage by the time full diffusion was reached. However, the behaviours trended towards an equilibrium production ratio of 1 : 1, as both yielded equivalent rewards.

Importantly, production did not simply lag behind knowledge acquisition—we observed divergences in the ordering of these events throughout the simulations when comparing the order of acquisition with the order of first-production (figure 2*b*). Beyond the ‘seed’ agent, we found large variation in these orders, with only 24.75% of agents producing the behaviour in the same order as acquiring knowledge of it (i.e. light diagonal pattern in figure 2*b*), and a divergence score of *d*_order_ = 1.95 [1.13, 2.71]. This can be interpreted as: after agent *i* acquired knowledge of the novel behaviour, approximately 2 more agents acquired knowledge of it before agent *i* first produced it. Simulations obtained a mean delay score of *d*_time_ = 5.33 [3.46, 7.13], representing the amount of time that passed between an agent acquiring a behaviour and producing it. We found that different production parameter settings could lead to substantial divergence between orders of acquisition and production. In such cases, the observed order of first-productions did not necessarily reflect the order of acquisition, and thus the underlying network structure. When measuring the *R*^2^ value of both orders, we found that under the reference constellation, *R*^2^ = 0.846, while under the constellation that produced the most divergence, *R*^2^ = 0.678 (figure 2*b*,*c*). Divergent orders could cause errors in NBDA inference, which we return to in §3b.

### (a) Production rules influenced tempo, divergence and delay

Our model yielded valuable insights into how rules that govern production choices influence diffusion dynamics. A sensitivity analysis showed that changes to almost any production parameter influenced diffusion dynamics, as measured by tempo, divergence and delay (further exploration of transmission parameters in electronic supplementary material, text S2 and table S1). Below we describe how varying the way agents made decisions influenced diffusion dynamics, detailed by parameter in table 2.

**Table 2.** Summary of results. Mean, standard error and 92% percentile interval (PI) for time-to-diffusion, time-to-first-production, divergence of orders of acquisition and first-production, and time delay between acquisition and first-production. The reference constellation of parameter values is given first, and rows are arranged by model parameters in the order presented in the main text.

	TTD		TTFP		divergence (*d*_order_)			delay (*d*_time_)	
value	mean ± s.e.	PI	mean ± s.e.	PI	% divergent	mean ± s.e.	PI	mean ± s.e.	PI
**reference constellation:** *σ* = 0.5, *f* = 1, *m* = 10, *ρ* = 0.1, *α* = 1									
	54.22 ± 1.15	[37.96,76.28]	61.11 ± 1.16	[44.96,85.04]	75.25	1.95 ± 0.05	[1.13,2.71]	5.33 ± 0.11	[3.46,7.13]
**social information bias (*σ*)**									
weak	41.18 ± 0.7	[30.96,55]	47.27 ± 0.75	[36.96,62.08]	71.17	1.82 ± 0.05	[0.78,2.78]	3.62 ± 0.07	[2.58,5.09]
medium	reference								
strong	82.86 ± 2.2	[56,124.2]	92.49 ± 2.18	[63,139.16]	77.33	2.17 ± 0.06	[1.39,3.39]	9.09 ± 0.19	[6.4,12.47]
**conformity bias (*χ*)**									
anti-conformist bias	46 ± 1.01	[31,65]	50.39 ± 1.1	[33,71.08]	68.25	1.56 ± 0.05	[0.78,2.43]	3.56 ± 0.07	[2.5,5.08]
linear bias	reference								
conformist bias	59.83 ± 1.48	[39.92,92.04]	72.24 ± 1.65	[48,107.08]	75.83	2.17 ± 0.06	[1.13,3.57]	6.43 ± 0.14	[4.28,8.88]
**memory (*m*)**									
10	reference								
20	68.93 ± 1.44	[44,95.16]	76.45 ± 1.52	[50.92,107]	69.92	1.65 ± 0.05	[0.87,2.61]	5.57 ± 0.12	[3.62,7.52]
30	83 ± 1.52	[61.96,108.24]	90.91 ± 1.62	[68,121.04]	68.50	1.47 ± 0.04	[0.78,2.35]	5.97 ± 0.12	[4.11,8.3]
**recent experience bias (*ρ*)**									
weak	56.63 ± 1.1	[40.96,75.04]	63.15 ± 1.11	[47,81.08]	72.92	1.9 ± 0.06	[1.12,2.96]	5.38 ± 0.12	[3.7,7.54]
medium	reference								
strong	50.26 ± 1.13	[34.96,69.12]	56.72 ± 1.16	[37.96,78]	76.29	2.09 ± 0.05	[1.38,3.13]	5.14 ± 0.11	[3.54,7.17]
**risk-appetite (*α*)**									
risk-tolerant	43.25 ± 0.89	[30.96,62.04]	47.84 ± 0.91	[35,68.04]	70.96	1.71 ± 0.05	[0.95,2.54]	3.56 ± 0.07	[2.46,4.88]
risk-neutral	reference								
risk-averse	99.48 ± 3.05	[57,164.04]	112.26 ± 3.07	[69.96,177]	79.29	2.41 ± 0.07	[1.3,3.57]	11.62 ± 0.22	[7.74,15.17]

**Figure 3.**
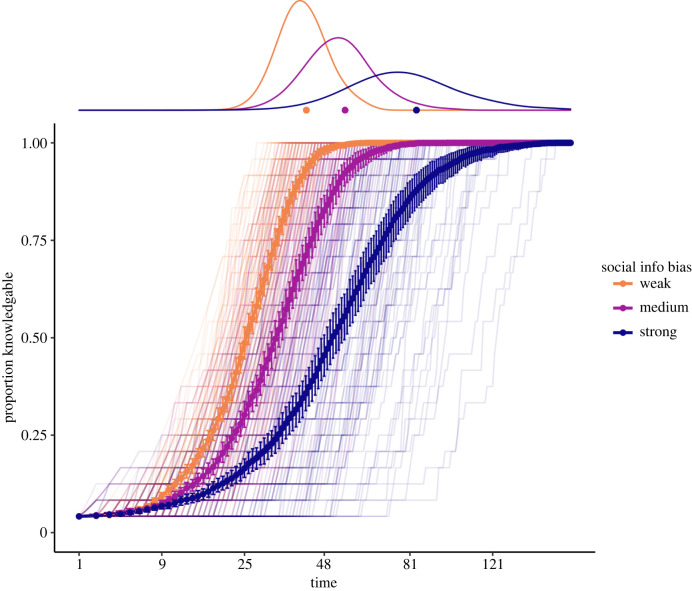
Strong social information bias slowed diffusion. Diffusion curves for the novel behaviour with distributions of time-to-diffusion at the top, pooled within social information bias (colour, mean with 95% bootstrapped CI and traces from individual simulations). Data shown for reference-level (300 simulations), and *x*-axis is square-root transformed for ease of reading. Strong social information bias within the sub-model of production greatly slowed diffusion. (Online version in colour.)

We first explored the effect of parameters that influenced how social information was used in production decisions. A weak social information bias (*σ*) resulted in a faster diffusion tempo with less divergence and delay compared with reference (equation (2.8)). Conversely, a strong *σ* slowed diffusion (figure 3; see electronic supplementary material, figure S3 for all parameters) and increased divergence and delay. This effect was explained by the large increases in delay between acquisition and production (*d*_time_ in table 2). When *σ* was strong, longer delays were obtained early in the diffusion, and when *σ* was weak, the delay remained consistent throughout the diffusion (electronic supplementary material, figure S4A). Agents with a weak *σ* were less influenced by the behaviour of associates, elevating the probability of producing the novel behaviour upon acquisition and consequently hastening its diffusion (electronic supplementary material, figure S5A).

We then explored the frequency-dependent production bias. Compared with reference, production conformity (*f* = 3) increased mean tempo, divergence and delay, while anti-conformity resulted in the opposite effects (table 2; electronic supplementary material, figure S3). Conformity consistently increased delay relative to intervals between acquisition events throughout diffusion (electronic supplementary material, figure S4B). Conformity amplified the social influence of naive associates, causing agents to prefer the established behaviour longer after acquisition (electronic supplementary material, figure S5B). This delayed the learning of the expected value of the novel behaviour, lowered production rates, and thus lowered the probability of further transmission.

Finally, relevant for both production and acquisition sub-models was the memory window (*m*). Increasing agents' memory slowed diffusion, yet resulted in less overall divergence (table 2; electronic supplementary material, figure S3B). This was because larger memory slightly increased delays, yet generated more consistent delay intervals throughout diffusion, while increasing intervals between acquisition events (electronic supplementary material, figure S4C). With a large memory, there was greater cultural inertia, as any single observation of the novel behaviour meant less to an agent, as it was outweighed by memories of many observations of the established behaviour (electronic supplementary material, figure S5C).

Next, we considered parameters relevant for personal experience. A strong bias towards recent experience (*ρ*) resulted in a faster tempo than reference, and reduced divergence and delay (table 2; electronic supplementary material, figure S3C). Larger *ρ* values hastened learning of the value of the novel behaviour, once acquired (electronic supplementary material, figure S5D). This made the novel behaviour more competitive against the established behaviour, as agents would be as likely to produce one as the other once their expected values were similar.

Finally, risk-averse agents (*α* = 0.5) greatly slowed tempo, increased divergence, and nearly doubled delay (table 2; electronic supplementary material, figure S3D), while risk-tolerant agents (*α* = 2) showed the reverse effects. In populations of risk-averse agents, delay decreased as the diffusion progressed as the productions of the novel behaviour by a growing number of knowledgeable agents offset the effect of risk-aversion (electronic supplementary material, figure S4E). Risk-averse agents, more sensitive to differences in value, preferred the established behaviour for longer periods after acquisition. Past experience had built up the expected value of the established behaviour, which exceeded that of the novel behaviour (electronic supplementary material, figure S5E). This decreased the competitiveness of the novel behaviour against the established behaviour.

In summary, changes in production rules that slowed diffusion (e.g. strong social information bias, conformity, risk-aversion) generally did so by decreasing the likelihood of production of the novel behaviour by newly knowledgeable agents. Any decrease in the likelihood of production was magnified by a feedback loop of reinforcement learning: lack of personal experience led to lower expectations of the novel behaviour, which encouraged the continued production of the established behaviour. Production rules that hastened diffusion (e.g. anti-conformity, strong bias towards recent experience and risk-tolerance) did so by increasing the relative competitiveness of the novel behaviour, once acquired.

### (b) The performance of inferential models when acquisition and production were linked

**Figure 4.**
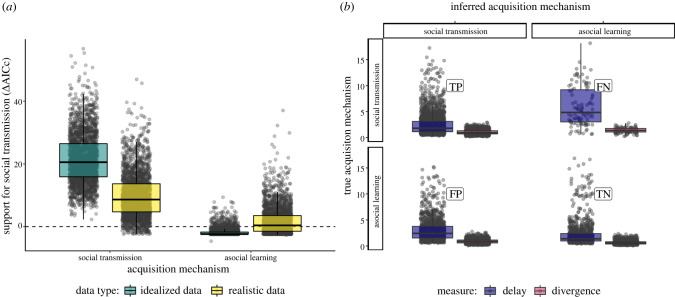
Delay and divergence correlated with false support in NBDA analyses. (*a*) Comparison of relative support for social transmission (ΔAICc) for idealized data (teal) and realistic data (yellow). Inference on idealized data matched our expectations, resulting in strong support for social transmission when social transmission was the generative mechanism behind the data, and support for asocial innovation when asocial innovation was the generative mechanism. However, using more realistic data, where transmission and production were linked, false support for the incorrect generative mechanism was more prevalent. (*b*) For realistic data, we compared delay and divergence measures from each simulation among true and false positives and negatives (label). Simulations that received the correct support had lower delay and divergence scores, compared with false positives and negatives. (Online version in colour.)

Continuous time-of-acquisition diffusion NBDA analyses of ‘ideal’ time-of-acquisition data yielded support for social transmission over asocial innovation as the acquisition mechanism for all social transmission simulations (median support ΔAICc: 20.5, figure 4*a*).

Under asocial innovation only, we found that support was ambivalent between asocial and social models (median ΔAICc: −2.38). The inferential models estimated the rate of social transmission close to that which had generated the data (electronic supplementary material, figures S6A,B). There was a trade-off between estimates for social transmission rate ($\hat{s}$) and base transmission rate ($\hat{\lambda_{0}}$), as simulation variance led to differences in how strictly the behaviour followed the network. In summary, these results mirrored previous results regarding the inferential properties of NBDA applied to acquisition data.

We then tested TADAc on more realistic first-production data, generated through linking the sub-models described in §2a and §2b. Generally, TADAc had more difficulty inferring the true acquisition mechanism. Out of 2430 simulations where social transmission was the true acquisition mechanism, the models estimated 102 false negatives (support for asocial innovation), and the distribution of support shifted towards asocial support (median ΔAICc: 8.65, figure 4*a*). The strongest drivers of false negatives were production conformity, present in 86% of all false negatives, followed by a strong social information bias and risk aversion, both present in 61% (electronic supplementary material, table S2). Out of 2430 simulations of asocial innovation, the models estimated 1318 false positives supporting social transmission instead, and the mean support shifted above 0 (median ΔAICc: 0.41). Strong social information bias and risk-aversion all increased the prevalence of false positives (electronic supplementary material, table S2). False support, positive or negative, was correlated with increased delays between acquisition and production, as well as more divergence between the order of acquisition and the order of first-production (figure 4*b*).

‘Ideal’ data, where all agents had the same repertoire over time, yielded point estimates of all parameters that were close to the true values used to generate the data. We then inferred parameter values from simulations where the novel behaviour diffused via social transmission, and again where the novel behaviour was asocially innovated. Summary statistics of parameter posteriors from all three conditions are summarized in electronic supplementary material, table S3, and visualized per condition in electronic supplementary material, figure S7. In simulations of heterogeneous repertoires with social transmission or asocial innovation, social information bias ($\hat{\sigma }$) was overestimated, with the true value not contained in the 92% HPDI for 179 of 180 simulations (electronic supplementary material, table S4). Recent experience bias ($\hat{\rho }$) was overestimated and the true value was not contained in the 92% HPDI in most cases (105/180 estimates). These results show that heterogeneous knowledge states during diffusions negatively affect the ability of EWA to infer learning parameters. In particular, the overestimation of $\hat{\sigma }$ was an artefact of agents appearing to be influenced by social information, simply because their knowledge state was constrained to the established behaviour.

## 4. Discussion

Understanding the relationship between psychological learning rules and population-level patterns is a long-standing research topic in cultural evolution [14,41,61,62]. We contribute to this understanding by underlining the difference between learning rules that influence production *contra* acquisition. In doing so, we have uncovered new predictions for how changes to learning rules affect cultural diffusion dynamics by altering the relative competitiveness of novel behaviours. Our work highlights how learning during behavioural maintenance, a type of internal cultural selection, [11,63,64], should be considered another dimension that operates alongside external selection between individuals via transmission biases [65–67] and transformation of behaviour via cognitive biases [68,69]. Not only does our model help to clarify discussions of strategies and biases, it has consequences for current practices in the field.

The case study of selective attrition in passerine birdsong illustrates why differentiating between acquisition and production matters [70–74]. Juvenile birds acquire song components from adult conspecifics, with an age-dependent transmission bias. However, as an adult, males preferentially produce the most frequently heard components, akin to a production conformity bias. This selective attribution then has consequences for repertoire size and composition (species with a fixed acquisition period [70–72]; open-ended learners [73,74]). Social learning strategies that occur either during acquisition or during production have often been treated as interchangeable [12–14]; our model facilitates clear identification of where strategies or biases occur. For example, conformity could be a transmission bias, such as when naive great tits disproportionately acquire the majority-produced solution to a foraging puzzle [24], or production bias, such as when chimpanzees switch preference to match the group [75]. Our model allows for the explicit definition of combinations of biases acting on either process. Prior models have focused on the evolution of transmission biases [61,76–79], for example, finding that conformist transmission should generally out-compete payoff-biased transmission [79]. We suggest that predictions might change if models allow for separate acquisition and production biases, as this creates dual optimization problems of repertoire and decision-making.

Our model assumed that in order to produce a behaviour, an individual must know that it can produce that behaviour, although it is not always easy to draw a line between acquisition and production. For example, many traits, including birdsong, are acquired through repeated observation and practice. However, our sub-model of acquisition was ambivalent towards the precise mechanism of how individuals acquire this knowledge [8], and could represent indirect social learning (e.g. enhancement) or more direct social learning (e.g. observational conditioning) [5]. Our model could therefore be interpreted as accounting for acquisition via practice, assuming that practice productions did not influence associates or the expected values of the practising agent. Our model could be further extended by implementing more complicated acquisition mechanisms (e.g. complex contagion [80]) or a function to define how rewards dynamically change with experience.

Our findings also have consequences for the way we analyse real-world data. We found that NBDA inference was less accurate in populations that were risk-averse, or highly sensitive to social information owing to increased divergence between orders of acquisition and production. Therefore, NBDA is perhaps best suited to larger, highly modular association networks, as longer intervals between acquisition events between clusters of nodes would help minimize divergence. While we did not conduct a thorough exploration of network features, the results from manipulating network architecture presented in electronic supplementary material, text S2 support our expectation that networks with particularly high local clustering and longer path lengths, characteristic of modular networks, can reduce the divergence between acquisition and production. In particular, ring lattice networks resulted in longer diffusions in which acquisition and production were less divergent. The influence of network size would further depend on how connectivity scales alongside size. All of these features merit future exploration.

We also demonstrated that social information bias in EWA measures only social influence, and cannot distinguish between social or asocial acquisition of novel behaviour. Since the label of 'social learning' has been given to both social transmission and social influence, this could lead those less familiar with EWA to misinterpret this parameter as evidence for social transmission. EWA might be of greater use when one can be relatively certain of homogeneous knowledge states. This includes the use of (1) experimental designs in which all choices are presented in a decision environment, (2) closed, rather than open-ended, tasks that constrain knowledge states and are less susceptible to morphological constraints, or (3) smaller social groups, where association is homogeneous and diffusion might occur rapidly relative to production rate. Finally, a recent study showed that a value-shaping reinforcement learning model more accurately reflected social information use in humans, compared with decision-biasing (as in EWA) [36]. This suggests a possibility for future improvement of the inferential EWA framework. Our study did not directly compare EWA with a value-shaping sub-model of production. However, as long as there is a mechanism that creates variation in the expected value of novel behaviour, we should still find an effect of production rules on diffusion dynamics.

In summary, we have shown how individual production decisions influence cultural diffusion dynamics at the population level. We rendered a clear distinction between acquisition and expression of behaviour, and thus social learning and social influence. We argue this distinction improves definitions of social learning biases, and highlights the important role of reinforcement learning in explaining patterns of cultural change. Finally, our work encourages further development of methods to account for divergences between acquisition and production, which may be much more commonplace than previously acknowledged.

## Data Availability

Code for statistical analyses, figures and agent-based models are available at https://github.com/michaelchimento/acquisition_production_abm (doi:10.5281/zenodo.6573285). Data are provided separately on Dryad (doi:10.5061/dryad.vx0k6djvk) [81]. Electronic supplementary material is available online [82].
